# Phytoconstituents Analysis and *In Vitro* Antiproliferative Activity of *Abrus precatorius* Leaves on Cancer Cells

**DOI:** 10.21315/tlsr2026.37.1.12

**Published:** 2026-03-31

**Authors:** Wan Suriyani Wan Ibrahim, Siti Farhanah Mohd-Salleh, Harishini Rajaratinam, Nor Amalia Nazri, John Ogbaji Igoli, Nurkhalida Kamal, Norzila Ismail

**Affiliations:** 1 Faculty of Bioengineering and Technology, Universiti Malaysia Kelantan, 17600 Jeli, Kelantan, Malaysia; 2 Department of Pharmacology, School of Medical Sciences, Universiti Sains Malaysia, 16150 Kubang Kerian, Kelantan, Malaysia; 3 Faculty of Medicine, Universiti Sultan Zainal Abidin, Medical Campus, 20400 Kuala Terengganu, Terengganu, Malaysia; 4 Department of Chemistry, Joseph Sarwuan Tarka University, PMB 2372 Makurdi, Nigeria; 5 Institute of Systems Biology (INBIOSIS), Universiti Kebangsaan Malaysia, 43600 Bangi, Selangor, Malaysia

**Keywords:** *Abrus precatorius*, Breast Cancer, GC-MS, Medicinal Plant, Phytochemical, Abrus precatorius, Kanser Payudara, GC-MS, Tumbuhan Berubat, Fitokimia

## Abstract

Traditionally in Malaysia, the leaves of *Abrus precatorius*, a flowering plant that belongs to the legume family, Fabaceae, are used to treat various ailments such as coughs, diarrhoea, wound healing and even as anticancer and antivirus remedy. The study aimed to identify the phytocompounds present in *A. precatorius* leaves using (1) different solvents and (2) two different extraction methods for each solvent. Additionally, we have also intended to investigate the anti-proliferative activity of those maceration-based extracts on selected cancer and normal cell lines. In this study, two extraction methods were used (maceration and Soxhlet extraction), using sequential solvents according to their polarity, starting with hexane, ethyl acetate and methanol. The phytocompounds were identified via the GC-MS technique. The findings reported that the ethyl acetate extract (obtained via Soxhlet extraction) contained the highest amount of terpenoids, while the methanol extract (obtained via maceration) contained the highest combination of terpenoids and phenolic compounds. The triple negative breast cancer cell line, MDA-MB-231 showed high sensitivity towards all *A. precatorius* leaf extracts (maceration-based). The hexane extract of *A. precatorius* leaves (maceration-based) showed the lowest IC^50^ on MDA-MB-231 cells at 80.75 ± 64.0 μg/mL. In conclusion, potential anticancer phytocompounds were identified in the *A. precatorius* leaves extracts such as 1-octacosanol, neophytadiene and 4-vinylphenol. All maceration-based extracts exhibited non-cytotoxicity effect on both normal breast cell MCF-10a and fibroblast cell NIH (3T3). The MDA-MB-231 cell line was the most sensitive towards all maceration-based extracts, particularly hexane-maceration based.

## Abstrak

Secara tradisional di Malaysia, daun Abrus precatorius, iaitu sejenis tumbuhan berbunga yang tergolong dalam keluarga kekacang, Fabaceae, digunakan untuk merawat pelbagai penyakit seperti batuk, cirit-birit, penyembuhan luka serta sebagai agen antikanser dan antivirus. Kajian ini bertujuan untuk mengenal pasti sebatian fitokimia yang terdapat dalam daun A. precatorius menggunakan (1) pelbagai jenis pelarut dan (2) dua kaedah pengekstrakan yang berbeza bagi setiap pelarut. Selain itu, kajian ini juga bertujuan untuk menilai aktiviti antiproliferatif ekstrak berasaskan kaedah maserasi terhadap beberapa garisan sel kanser dan sel normal terpilih. Dalam kajian ini, dua kaedah pengekstrakan telah digunakan, iaitu kaedah maserasi dan pengekstrakan Soxhlet, dengan menggunakan pelarut secara berjujukan berdasarkan kepolarannya, bermula dengan heksana, diikuti etil asetat dan metanol. Sebatian fitokimia dikenal pasti menggunakan teknik kromatografi gas–spektrometri jisim (GC-MS). Hasil kajian menunjukkan bahawa ekstrak etil asetat (diperoleh melalui pengekstrakan Soxhlet) mengandungi jumlah terpenoid tertinggi, manakala ekstrak metanol (diperoleh melalui kaedah maserasi) mengandungi gabungan terpenoid dan sebatian fenolik tertinggi. Garisan sel kanser payudara triple negatif, MDA-MB-231, menunjukkan sensitiviti yang tinggi terhadap semua ekstrak daun A. precatorius berasaskan kaedah maserasi. Ekstrak heksana daun A. precatorius (berasaskan maserasi) menunjukkan nilai IC_50_ terendah terhadap sel MDA-MB-231, iaitu 80.75 ± 64.0 μg/mL. Kesimpulannya, beberapa sebatian fitokimia berpotensi sebagai agen antikanser telah dikenal pasti dalam ekstrak daun A. precatorius, seperti 1-oktacosanol, neofitadiena dan 4-vinilfenol. Semua ekstrak berasaskan kaedah maserasi menunjukkan kesan tidak sitotoksik terhadap kedua-dua sel payudara normal MCF-10a dan sel fibroblas NIH (3T3). Garisan sel MDA-MB-231 merupakan yang paling sensitif terhadap semua ekstrak berasaskan kaedah maserasi, khususnya ekstrak heksana berasaskan maserasi.

**HIGHLIGHTS**

- The ethyl acetate extract (obtained via Soxhlet extraction) of *Abrus precatorius* leaves contained the highest amounts of terpenoids, while the methanol extract (obtained via maceration) of *A. precatorius* leaves contained the highest combination of terpenoids and phenolic compounds.
- The triple negative breast cancer cell line, MDA-MB-231 showed high sensitivity towards all *A. precatorius* leaf extracts (maceration-based) and the hexane extract of *A. precatorius* leaves (maceration-based) showed the lowest IC^50^ on MDA-MB-231 cells at 80.75 μg/mL.
- Potential anticancer phytocompounds were identified in the extracts of *A. precatorius* leaves such as 1-octacosanol, neophytadiene and 4-vinylphenol.

## INTRODUCTION

*Abrus precatorius* is a flowering plant that belongs to legume family, Fabaceae (Vijayan & Thirumal 2025). The common names of *A. precatorius* include jequirity, Crab’s eye, Rosary pea, precatory pea or bean, John crow bead, Indian liquorice, *Akar saga* and jumble bead. Decoction of the *A. precatorius* leaves is widely practised as a treatment for colds, cough and colic (Solanki & Zaveri 2012; Okhale & Nwanosike 2016). A mixture of rice starch and leaf paste is consumed orally for anthrax treatment (Pokharkar *et al*. 2011). Powdered leaves paste is used for conjunctivitis and convulsion in children (Joshi & Tyagi 2011). Phytochemical analysis of the leaves and roots of *A. precatorius* demonstrated the presence of glycyrrhizin (Karwasara *et al*. 2010), an important compound of liquorice (Killacky *et al*. 1976; Račková *et al*. 2007), which is widely used in the food and pharmaceutical industry. A known triterpenoid and three novel triterpenoids were identified from the acid-hydrolysed methanol-soluble leaves extract of *A. precatorius* (Kim *et al*. 2002). From the *n*-butanol leaves extract of *A. precatorius*, other compounds identified were abrusoside A (Choi *et al*. 1989), abrusosides B, C, D, plus three other sweet glycosides based on the novel cycloartane-type aglycone, abrusogenin (Kinghorn & Soejarto 2002). Metabolites of *A. precatorius* are reported to target multiple oncogenic and onco-suppressive signalling for cancer prevention and intervention (Kaur *et al*. 2022).

Medicinal plants are gaining worldwide recognitions because of their diversity and broad pharmacological activities from their therapeutic phytochemicals (Mustafa *et al*. 2017; Alam *et al*. 2022). In general, phytochemicals are extracted from different parts of plants like seeds, seed coats, barks, leaves, flowers, pulps, roots and shoots. The extraction of the phytochemical compounds is significant in the exploration of new therapeutic biomolecules that could potentially serve as medicinal agents. To date, thousands of phytochemical compounds have been reported to have beneficial biological activities such as antimicrobial, antioxidant, anticancer, etc. Phenolic compounds and flavonoids, for instance, have a great impact on health and cancer prevention (Venugopal & Liu 2012).

Optimising the extraction procedure is the most crucial part in studying the properties of medicinal plants. Extraction is a standard procedure to separate phytochemical compounds using selective solvents. Decoction and maceration are amongst the commonly used techniques in traditional practices, while the Soxhlet extraction is more distinguished in the industrial counterpart. Maceration is a technique adopted from wine making where plant materials are soaked in a closed container with a solvent for at least three days at room temperature (Handa 2008). Soxhlet extraction or also known as continuous hot extraction uses the Universal Extraction System (Büchi) (Ramluckan *et al*. 2014), where heated solvent in a flask, vaporises into the thimble containing grounded plant material, condenses in the condenser and drip back into the hot flask. This process is repeated until the colour of the solvents becomes clear.

Plant extracts encompass numerous phytochemical compounds, which pose a challenge in order to separate and identify them due to their polarity. Chromatography is a process to separate any molecules based on their shape, size and charge. With the advancement of research and technologies, different separation techniques have been introduced to identify and isolate these compounds such as gas chromatography (GC), paper chromatography, thin layer chromatography (TLC), high-performance thin layer chromatography (HPTLC), column chromatography, overpressure layer chromatography (OPLC) and high-performance liquid chromatography (HPLC) (Attimarad *et al*. 2011; Tyihák *et al*. 2016). GC is a technique used to separate volatile compounds, where the liquid phase is separated from the gas phase. It is one of the most important analytical methods in organic chemical analysis to determine individual substances in complex mixtures. Mass-spectrometry is an analytical method that measures masses within a sample by ionising the chemical species and sorting their ions based on the mass-to-charge ratio (Agarwal & Goyal 2017). This detection method provides meaningful data by determining the substance molecules or fragments directly. Therefore, the integration of gas-chromatography and mass-spectrometry into a single GC-MS system has been a great platform for many laboratories to run a quantified detection analysis due to its high selectivity and very high sensitivity (Belwal *et al*. 2018).

We have previously reported on the phytocompounds present in the aqueous extract (maceration-based) of *A. precatorius* leaves using the GC-MS technique (Wan-Ibrahim *et al*. 2018). As an extension to our earlier investigation, we have further addressed the phytocompounds present in *A. precatorius* leaves extracted using:

1. different solvents,
2. two different extraction methods (via maceration and using the Soxhlet method) for each solvent, and
3. the identification of phytocompounds using the GC-MS technique.

Additionally, we have also intended to investigate the anti-proliferative activity of the maceration-based extracts in selected cancer and normal cell lines. Previously, we have studied the anti-proliferative activity of *A. precatorius* leaf extracts using hexane, ethyl acetate, methanol and aqueous solvents, which were prepared via the Soxhlet method, on selected cancer and normal cell lines (Wan-Ibrahim *et al*. 2019). It was noted that the methanolic extract of *A. precatorius* leaves (Soxhlet-based extraction) exhibited the lowest IC_50_ value against MDA-MB-231 cells at 26.40 μg/mL (Wan-Ibrahim *et al*. 2019). Thus, the inclusion of the IC_50_ values of the hexane, ethyl acetate and methanol extracts of *A. precatorius* leaves, prepared via maceration technique, completes our final investigation on the *in vitro* anti-proliferative activity of *A. precatorius* leaves on cancer and normal cell lines.

## MATERIALS AND METHODOLOGY

### Plant Collections

*A. precatorius* leaves were collected from Kampung Sabak, Pengkalan Chepa, Kelantan, Malaysia (102.315141). It was authenticated by Dr. Rahmad Zakaria from the Herbarium Unit, School of Biological Sciences, Universiti Sains Malaysia and the voucher specimen (USM 11730) has been submitted for future references.

### Preparation of Leaf Sample

The leaves of *A. precatorius* were collected, cleaned and oven-dried at 50°C, and then ground to powder with a mechanical grinder.

### Maceration of the Leaves by Hexane, Ethyl Acetate and Methanol Solvents

Three successive extractions of the *A. precatorius* leaves by maceration were conducted. Approximately 18 g of dried powdered leaves were soaked in three different solvents successively for about one month each. The leaves were macerated in hexane, then in ethyl acetate, and finally in methanol. After each session, the mixture of the leaves and solvent was filtered then the filtrate was left to dry under the fume hood to obtain the dried crude extract. The remaining leaves were also placed under the fume hood to allow the solvent to evaporate before macerating with the next solvent.

### Successive Solvent Soxhlet Extraction

About 22 g of ground *A. precatorius* powder was subjected to successive Soxhlet (Büchi) extraction with hexane, ethyl acetate and methanol. Upon completion of the first extraction with hexane, the solution was dried using a rotary evaporator. The remaining powdered leaves in the thimble was left to dry overnight in fume hood to evaporate residual hexane. Then subsequent extraction with ethyl acetate was performed in the same manner and followed by methanol. All extracts were kept in −20°C until further used.

#### Gas Chromatography–Mass Spectrometry (GC-MS)

Hewlett Packard 6890 Gas Chromatograph with 5973N Mass Selective Detector was used to carry out the GC-MS. The column was a fused silica capillary, HP-5 column (30 m × 0.25 mm i.d × 0.25 μm film thickness) (Agilent Technologies, USA). The carrier gas was helium with flow rate at 1.0 mL/min with the oven temperature programmed from 50°C (held for 2 min) to 280°C (held for 10 min) at a rate of 20°C/min. The injection and interface temperatures were set at 250°C and 280°C, respectively. One microliter sample was injected in split less mode and was analysed in MS full scan mode (*m/z* 40–650). The electron ionisation was fixed at 70 eV. Acquisition of data was performed using the Chemstation software.

#### Identification of phytochemical compounds

The mass spectrum of the GC-MS was interpreted against the database of the National Institute of Standards and Technology (NIST02) and Wiley275 libraries with matches of ≥ 80% to identify phytochemical compounds.

#### Cell culture of cancer and normal cell lines

The cervical (HeLa), breast (MCF7 and MDA-MB-231), and colon (SW 480) cancer cell lines, and normal breast (MCF-10a) and mouse fibroblast (NIH (3T3)) cell lines were obtained from the American Type Culture Collection (ATCC, USA). The cells were stable as they were not mixed with other tissues, hence enabling us to produce more consistent results for comparison. A complete medium containing Dulbecco’s Modified Eagle’s Medium (DMEM, Gibco), 10% of foetal bovine serum (FBS, Gibco), 1% of penicillin-streptomycin (Gibco) under a humidified air atmosphere containing 5% carbon dioxide (CO_2_) at 37°C were used in the culture of the cell lines (Mohd-Salleh *et al*. 2019).

By including cell lines from different types of cancers (cervical, breast and colon), the study was able to assess the broad-spectrum efficacy of the tested extracts. The use of MCF7 and MDA-MB-231 addressed both hormone sensitive and hormone insensitive breast cancers, thus providing a comprehensive understanding on the extract’s effects on distinct molecular subtypes. Including MCF-10a and NIH (3T3) cell lines enables evaluation of the extract’s selectivity for cancer cells over normal cells. This is crucial for determining the therapeutic potential and minimising side effects.

### Cytotoxic Activity Assay

The screening of the anti-proliferative activity of *A. precatorius* leaves extracts (maceration-based) was carried out via the MTT (3-[4,5-dimethyl thiazol-2-yl] 2,5-diphenyl tetrazolium bromide) assay and was performed on both cancer and normal cell lines. The screening of the anti-proliferative activity of *A. precatorius* leaves extracts (Soxhlet-based) was previously carried out by our team using the MTT assay and the IC_50_ values for the anti-proliferative activity of the Soxhlet-based extracts of *A. precatorius* leaves, using hexane, ethyl acetate, methanol and aqueous solvents were published (Wan-Ibrahim *et al*. 2019).

Cells were seeded into 60 wells at the centre of a 96-well plate with a concentration of 5 × 10^4^ cells/mL per well for all cell lines. Wells at the edges of the plate were filled with water to prevent the plate from drying during the incubation. The next day the media was discarded, then 200 μL of fresh media were added into the wells. Extracts of *A. precatorius* leaves were added following a 1:2 serial dilution starting from 495 μg/mL until 1.9 μg/mL in each well. Following 72 h of incubation, the media containing the extracts were discarded and cells were washed three times with 1× PBS. Then, the cells were incubated with MTT reagent for 4 h. The reaction was stopped with the addition of 100 μL of DMSO. Absorbance was read at an optical density of 570 nm. The percentage of cell viability was determined according to the following equation.


$$\text{Percentage of cell viability }(\%)=\frac{\text{Absorbance of treated cells }(\text{extracts or Tamoxifen})}{\text{Absorbance of treated cells }(\text{DMSO})}\times 100$$

Once the cytotoxicity assay was completed and the percentages of cell viability were recorded, a sigmoidal dose response curve was plotted to determine the IC_50_ via the Microsoft Excel software. The IC_50_ represents the concentration of a drug or compound that inhibits cell viability (or activity) by 50%.

### Statistical Analysis

Data are expressed in mean ± standard deviation (SD) from three independent experiments (triplicates) to represent the IC_50_ values.

## RESULTS

### Maceration of the Leaves (Hexane)

A total of 36 compounds were identified in this extract, listed in Table 1. The main compounds identified in this hexane leaves extract by maceration were 1-octacosanol (24.09%), 1-heptacosanol (21.80%) and oxirane heptadecyl- (20.85%). Twelve compounds were identified under the terpenoids group.

### Maceration of the Leaves (Ethyl acetate)

A total of 21 compounds were identified, as listed in Table 2. The main compounds identified in this extract were 2-hexadecene,3,7,11,15-tetramethyl-(R-(R*,R*-E)- (16.02%), octacosyl acetate (8.67%) and phytol (7.60%).

### Maceration of the Leaves (Methanol)

A total of 21 compounds were identified as listed in Table 3. The main compound group in this extract was phenolic compounds (3.44%), consisting of phenol, 4-vinylguaiacol, syringol, methylparaben and 4-methyl-2,5-dimethoxybenzaldehyde. Cyclotetracosane was the most abundant compound in this extract.

### Soxhlet Extraction of the Leaves (Hexane)

A total of 22 compounds were identified from this extract, as listed in Table 4. The main compound identified from this extract was oxirane, hexadecyl- at 15.72%, followed by 1-eicosanol at 10.53%.

### Soxhlet Extraction of the Leaves (Ethyl acetate)

Neophytadiene (32.56%) was the main compound identified in this extract. The total of 37 compounds were identified from this extract, as listed in Table 5.

### Soxhlet Extraction of the Leaves (Methanol)

A total of 29 compounds were found in this extract as listed in Table 6. Neophytadiene (12.18%) and 4-vinylphenol (12.18%) were the two main compounds identified.

### Cytotoxicity Activity Assay of A. precatorius Leaves Extracts

Table 7 displays the IC_50_ values of *A. precatorius* leaf extracts (maceration-based) against selected normal and cancer cell lines. The lowest IC_50_ value was exhibited by the hexane extract on breast cancer cell line, MDA-MB-231, at 80.75 μg/mL, and all extracts showed no cytotoxicity on both normal cell lines, MCF-10a, even at the highest concentration of 495 μg/mL. The proliferation (cell viability) plots for each cell line are included in the Appendix.

## DISCUSSION

In this study, two extraction methods were used for the successive solvent extractions, which were Soxhlet and maceration. Soxhlet extraction applied heat in a shorter time while maceration involved prolonged soaking without heat. Successive solvent extraction meant the leaves were extracted first using hexane, ethyl acetate and then with methanol, in the manner of increasing polarity index (P′). Burdick and Jackson have arranged and listed solvents in order of increasing P′ (Barwick 1997). Hexane has a P′ of 0.1, P′ for ethyl acetate is 4.4 and P′ for methanol is 5.1. Meanwhile, aqueous has the highest P′ at 10.2. Low P′ solvent will extract higher volatile compounds, while the higher the P′, the less volatile compounds will be extracted. Soxhlet extraction and maceration differed in the types and abundance of metabolites extracted, reflecting the influence of temperature and solvent-sample contact (Zhang *et al*. 2018). The use of hexane, ethyl acetate and methanol solvents in extracting the compounds in *A. precatorius* leaves was based on their different polarities, which selectively solubilise different classes of phytochemicals. Hexane targets non-polar constituents, ethyl acetate efficiently extracts semi-polar compounds such as terpenoids, whereas methanol solubilises polar metabolites such as phenolics and flavonoids (Lee *et al*. 2024). This polarity-guided extraction strategy enabled a comprehensive characterisation of leaf metabolites. This is essential for assessing their individual anticancer properties.

1-octacosanol was the main compound identified in hexane-maceration based extract. This compound is a fatty acid alcohol mostly found in waxes of leaves, and it is chemically similar to vitamin E. To be beneficial for health, this compound must be considered as a supplement because only a small amount of it can be included in the diet (Taylor *et al*. 2003). Red-coloured rice of the Korean rice genotype presented highest antioxidant activity and contained the highest level of octacosanol (Cho *et al*. 2017). Though not scientifically proven, this compound is used as a supplement for many things including Parkinson disease, managing high cholesterol and atherosclerosis, improving athletic performances and also for amyotrophic lateral sclerosis (Walsh *et al*. 2016). A study recently published showed that combined supplement with addition of 1-octacosanol can physically boost the fitness of dogs trained for drug-detection (Menchetti *et al*. 2019). Another fatty alcohol highly presented in hexane-maceration extract was heptacosanol (21.80%). However, only 0.24% of the fatty alcohol was identified in the hexane Soxhlet extract. No health benefits or bioactivity has been associated with this compound. 1-eicosanol was another fatty alcohol highly presented in the hexane Soxhlet extract which was mainly used as an emollient in the cosmetics industry [PubChem CID=12404] (Kim *et al*. 2019).

Neophytadiene was the main compound found in the ethyl acetate Soxhlet extraction. It is an antioxidant compound known for its biological activity as anti-inflammatory, antipyretic, good analgesic and antimicrobial. Neophytadiene belongs to a group of compounds known as sesquiterpenoids, which consist of terpenes containing three consecutive isoprene units (Kalaiselvi *et al*. 2018). This compound was not found in the ethyl acetate maceration extract. The main compound found in the ethyl acetate maceration extract was 2-hexadecene,3,7,11,15-tetramethyl-(R-(R*,R*-E)-. Neophytadiene presence was very little with a peak area of 1.54% in hexane Soxhlet extract, and 1.23% in hexane-maceration extract. This compound was also one of the main compounds identified in the methanol Soxhlet extract besides hexadecenoic acid, methyl ester.

Hexadecanoic acid, methyl ester, also known as methyl palmitate belongs to a group of fatty acid methyl ester (Al-Saadi *et al*. 2017). This compound has been reported to significantly induce dilation in aorta (Wang *et al*. 2018), reduce the levels of tumour necrosis factor-alpha (TNF-α), interleukin-10 (IL-10) and prostaglandin E2 (PGE2) without jeopardising the levels of ATP in cells. Besides, methyl palmitate is also reported to inhibit nitric oxide production and phagocytic activity of certain cells (Sarkar *et al*. 2006; Wang *et al*. 2010; Wang *et al*. 2018). Methyl palmitate is also known as vasodilator which enhances blood flow in cerebral and promote neuronal cell survival after cardiac arrest (Lee *et al*. 2019). This therapeutic potential of methyl palmitate would lead to improvement of functional learning and memory subsequent of cardiac arrest-induced brain injury (Lee *et al*. 2019).

In this current study, extract from the ethyl acetate and methanol obtained by Soxhlet exhibited the highest phenolic and terpenoid compounds. 4-vinylphenol was the highest phenolic compound identified in the methanol extract (Soxhlet) and neophytadiene was the highest terpenoid compound identified in that extract. Cirsimaritin is another phenolic compound identified in both ethyl acetate and methanol (Soxhlet) extracts. This compound inhibited nitric oxide production and exhibited anti-inflammatory activity (Shin *et al*. 2017). Another compound detected in the ethyl acetate (Soxhlet) profile is 3-Methoxy-4,5,7-trihydroxyflavone, also known as chrysoeriol. This compound is derived from luteolin, another phytocompound widely studied in medicinal plants. Recently, it was found that this compound exhibited anti-inflammatory (Limboonreung *et al*. 2020) and anticancer activity (Wei *et al*. 2019).

Phytochemicals found in plants are generally known as primary and secondary compounds. Primary compounds are generally present as the building blocks of plants which includes sugars, proteins, and chlorophyll. Secondary compounds include phenolic compounds, alkaloids, terpenoids, steroids and many more (Wadood *et al*. 2013). The biggest group of phytochemicals is the phenolic compounds, and most of these compounds are found in plant-based foods, mainly fruits and vegetables, such as cherries, grapes, citruses, tomatoes, apples, peaches and berries (Basli *et al*. 2017). Phenolic compounds are widely studied for its health benefits, especially the ability to exhibit as an anticancer agent. This ability might be attributed to the antioxidant activity posed by phenolic compounds. Oxidative stress is one of the causes of cancer occurrences. The chemo-preventive structures in phenolic compounds are able to induce cell cycle arrest thus inhibiting DNA binding and proliferation and regulate the expression of ontogenesis and carcinogen metabolism (Huang *et al*. 2009). Naringenin, a phenolic compound identified in the ethyl acetate extract (Soxhlet), exhibited cytotoxic effect on colon carninoma. In this particular study, naringenin was isolated from the citrus (Song *et al*. 2016).

Terpenoids is also another compound of interest which have been identified to demonstrate the anti-proliferative activity on cancer cells. Subclasses of terpenoids are believed to contribute as anticancer agents include monoterpenoid, diterpenoid, triterpenoid and sesquiterpenoid (Huang *et al*. 2012). In our current study, *A. precatorius* leaves extract from ethyl acetate Soxhlet extraction presented the highest terpenoids at 36.97%. While the methanol Soxhlet extraction showed the highest phenolic compounds presented at 13.88% and terpenoids at 14.08%. Although there are a lot of therapeutic potential of the compounds identified in all extracts, our study is focusing on compounds promoting anticancer activity.

In the present study, compared to cervical and colon cancers, breast cancer was the most vulnerable towards the anti-proliferative activity of the *A. precatorius* leaf extracts (maceration-based) as indicated by the lowest IC_50_ recorded in the MDA-MB-231 cell line. When comparing the subtypes of breast cancer, it was evident that MDA-MB-231 cells that is generally known as triple-negative breast cancer which is hormone insensitive (Simu *et al*. 2021) was more vulnerable to the anti-proliferative activity of maceration-based extracts of *A. precatorius* leaves, compared to MCF7 which is a hormone sensitive (Comşa *et al*. 2015) breast cancer cell line. Such finding highlights the role played by molecular subtypes of breast cancer in responding to the anti-proliferative mechanism of *A. precatorius* leaves. In agreement, Sofi *et al*. (2012) reported that *A. precatorius* leaf extract exhibits a growth inhibitory effect by inducing apoptosis in MDA-MB-231 cells. In 2018, Sofi *et al*. identified stigmasterol hemihydrate and β-monolinolein as the two main cytotoxic constituents of leaf extract of *A. precatorius*, with an IC_50_ value of 74.2 μg/mL and 13.2 μg/mL, respectively, in MDA-MB-231 cells. On normal breast cell line, MCF-10a, and normal fibroblast cell line, NIH (3T3), the maceration-based extracts failed to display any anti-proliferative activity at the maximum concentration of 495 μg/mL as shown in Table 7.

The hexane extract of *A. precatorius* leaves (maceration-based) showed the lowest IC_50_ on MDA-MB-231 cells at 80.75 μg/mL. With 1-octacosanol being the main compound identified in hexane-maceration based extract, we believe that the compound might be responsible for the anti-proliferative activity of the hexane-maceration extract. Based on a review by Zhou *et al*. (2022), it was noted that octacosanol possessed anti-hypoxia, anti-oxidation, anti-inflammation and anti-tumour properties. Octacosanol extracted from *Tinospora cordifolia* inhibited the proliferation and metastasis of *Ehrlich ascites* tumour cells *in vivo*, via the reduction of VEGF generation and the activity of matrix metalloproteinases (MMPs), thus, blocking the transcription factor NF-κB (Thippeswamy *et al*. 2008). Chu *et al*. (2016) confirmed that octacosanol could exhibit anti-tumour effects by inhibiting the activity of MMPs and the translocation of NF-κB.

The variations in IC_50_ activity between the different extracts (hexane, ethyl acetate and methanol) prepared via maceration method could be attributed to the different bioactive compounds and concentration of the compounds in the extracts. The hexane extract (maceration-based) showed low IC_50_ readings when tested on MDA-MB-231 and SW480 cancer cell lines compared to ethyl acetate and methanol extracts prepared using similar method. Compounds such as 1-octacosanol (Saenthaweesuka *et al*. 2022; Jia *et al*. 2025), oxirane heptadecyl (Nasiruddin *et al*. 2022) and 1-heptacosanol (Chella Perumal *et al*. 2015), detected in the hexane extract (maceration-based) were found to exhibit anticancer properties. In contrast, the ethyl acetate extract mainly consists of 2-hexadecene,3,7,11,15-tetramethyl-(R-(R*,R*-E), which showed no concrete evidence of its effectiveness against cancer cells. On the other hand, the methanol extract displayed the presence of cyclotetracosane. Mongalo *et al*. (2019) reported that cyclotetracosane was found in the GC-MS analysis of *Jatropha zeyheri* roots extract, which was effective against colon adenocarcinoma. However, the concentration of the cyclotetracosane was slightly lower compared to the other compounds discussed in their respective extracts. An important point to note is that the amount of 1-octacosanol (0.07% of area) was less abundant in the methanol extract compared to its presence in the hexane extract (24.09% of area) prepared via maceration. Such comparison may help explain the difference in the IC_50_ readings between the three extracts of *A. precatorius* leaves prepared via maceration.

However, the Soxhlet-based extraction of *A. precatorius* leaves conducted using the hexane, ethyl acetate and methanol solvents exhibited better anti-proliferative activity against MCF7 and MDA-MB-231, with lower IC_50_ values (Wan-Ibrahim *et al*. 2019) compared to the anti-proliferative activity of maceration-based extracts, currently reported in this study (Table 7). The IC_50_ values of the *A. precatorius* leaf extracts (Soxhlet-based), prepared using hexane, ethyl acetate and methanol solvents were 52.65 μg/mL, 99.00 μg/mL and 59.03 μg/mL, respectively for MCF7 cells, and 45.60 μg/mL, 54.50 μg/mL and 26.40 μg/mL, respectively for MDA-MB-231 (Wan-Ibrahim *et al*. 2019). The methanolic extract of *A. precatorius* leaves (Soxhlet-based extraction) showed the lowest IC_50_ on MDA-MB-231 cells at 26.40 μg/mL (Wan-Ibrahim *et al*. 2019). We postulate that 4-vinylphenol (phenolic) and neophytadiene (terpenoids) identified in the methanolic extract might be responsible for the anti-proliferative activity of the methanolic extract of *A. precatorius* leaves (Soxhlet-based extraction) reported in our previous investigation. In agreement, Leung *et al*. (2018) reported that 4-vinylphenol inhibits metastasis and cancer stemness in breast cancer cells. Furthermore, the minor presence of neophytadiene in the hexane-maceration extract of *A. precatorius* leaves may also contribute to the anti-proliferative activity of MDA-MB-231 reported in the current study. Recently, Selmy *et al*. (2023) conducted an *in silico* study which showed that neophytadiene blocked three receptors which have main role in cancer viability.

In terms of limitations, our study did not address the plausible interactions between the bioactive compounds of the hexane extract of *A. precatorius* leaves (maceration-based), which showed the lowest IC_50_ on MDA-MB-231 cells. The hexane extract (maceration-based) was found to be rich in 1-octacosanol and 1-heptacosanol. Polyethylene glycol (PEG)-derivatised octacosanol has been shown to self-assemble into micelles that effectively encapsulate paclitaxel and docetaxel, improving delivery and anti-tumour activity in preclinical models (Chu *et al*. 2016; Chen *et al*. 2022). These studies may provide a theoretical hint that octacosanol in our hexane fraction could plausibly alter the pharmacokinetics and delivery of lipophilic chemotherapeutics or compete for metabolic and transporter pathways for lipophilic compounds, considering that octacosanol and heptacosanol are long-chain fatty alcohols, which are strongly lipophilic. Direct mechanistic studies involving isolated forms of these bioactive compounds and breast cancer drugs are scarce and warrant further investigation through targeted combination assays, metabolic studies and drug-transport studies.

## CONCLUSION

The phytocompounds present in *A. precatorius* leaves extracted using hexane, ethyl acetate and methanol solvents were identified using the GC-MS technique. Two different extraction methods (maceration and using the Soxhlet method) employed for each solvent produced a diverse array of phytocompounds that are different from each other. The anti-proliferative activity of the extracts (based on the maceration extraction) exhibited non-cytotoxicity effect on both normal breast cells, MCF-10a and fibroblast cells, NIH (3T3). The MDA-MB-231 cell line was the most sensitive towards all maceration-based extracts, regardless of solvent. The hexane extract of *A. precatorius* leaves (maceration-based) showed the lowest IC_50_ on MDA-MB-231 cells. The anti-proliferative activity of the hexane extract could be due to the presence of phytocompounds such as 1-octacosanol and neophytadiene (terpenoids), which have anticancer properties. These findings provide a better understanding of *A. precatorius* leaves as potential source of anticancer agents.

## Figures and Tables

**TABLE 1:** Compounds present in the hexane extract (prepared via maceration technique) of *A. precatorius* leaves using GC-MS.

Retention time (min)	Name of compound	Area (%)
	**Terpenoids**	
7.114	Dihydromyrcenol	0.01
7.387	Linalool	0.02
8.417	Citronellol	0.01
8.109	m-methylacetophenone	0.01
8.368	β-cyclocitral	0.01
8.627	β-cyclohomocitral	0.01
9.243	Naphtalene,1,2,3,4-tetrahydro-1,1,6-trimethyl-	0.02
9.733	Geranyl acetone	0.02
9.936	β-lonone	0.07
11.512	Neophytadiene	1.23
14.894	Squalene	0.91
14.957	Geranylgeraniol	0.13
	**Others**	
5.938	3-octanone	0.01
10.195	2(4h)-benzofuranone,5,6,7,7a-tetrahydro-4,4,7aa-trimethyl	0.15
10.734	Methyl dihydrojasmonate	0.20
11.162	Octanal, 2-(phenylmethylene)-	0.08
13.339	Ethyl eicosanoate	0.18
13.451	4,8,12,16-tetramethylheptadecan-4-olide	0.20
13.563	Tetracosane	1.00
13.815	Butyl 9,12-octadecadienoate	0.14
13.885	Eicosane	0.32
13.955	2-Monopalmitin	0.66
14.151	4-methyl-1-anthracenamine	0.08
14.305	16-heptadecenal	0.16
14.495	Heptadecane	1.55
14.586	Tetracosanoic acid, methyl ester	0.80
15.076	Octadecane, 1-chloro-	1.98
15.363	Tridecane	0.86
15.531	1,19-eicosadiene	7.98
15.839	1-heptacosanol	21.80
15.923	Octadecanal	0.78
15.972	16-octadecenal	1.07
16.441	Oxirane heptadecyl-	20.85
16.868	1-octacosanol	24.09
17.582	Dl-α-tocopherol	0.62
19.585	Cyclotriacontane	0.20

**TABLE 2:** Compounds present in the ethyl acetate extract (prepared via maceration technique) of *A. precatorius* leaves using GC-MS.

Retention time (min)	Name of compound	Area (%)
	**Phenolic compounds**	
8.984	2-Methoxy-4-vinylphenol	0.72
	**Terpenoids**	
12.59	Phytol	7.60
14.886	Squalene	0.73
	**Steroids**	
16.28	7-ergosterol	2.06
16.805	β-Sitosterol	1.78
	**Others**	
7.121	1-Ethyl-2-pyrrolidinone	0.01
8.41	Coumaran	0.12
9.229	Naphthalene,1,2-dihydro-2,5,8-trimethyl-	0.09
9.474	1-(3,6,6-Trimethyl-1,6,7,7a-tetrahydrocyclopenta[c]pyran-1-yl)ethanone	0.06
11.519	2-hexadecene,3,7,11,15-tetramethyl-(R-(R*,R*-E)-	16.02
11.855	Methyl hexadecanoate	1.70
12.786	Ethyl linolenate	0.18
13.941	2-Palmitoglycerol	1.88
14.515	Nonanoic acid,9-(3-hexenylidenecyclopropylidene)-,2-hydroxy-1-(hydroxymethyl)	5.03
15.055	Cyclotriacontane	2.52
15.244	D-δ-tocopherol	2.54
15.902	Vitamin E	2.05
16.133	Z-14-Nonacosane	2.48
16.574	Octacosyl acetate	8.67
17.141	Triacontyl acetate	2.44
17.764	Cyclotriacontane	1.08

**TABLE 3:** Compounds present in the methanol extract (prepared via maceration technique) of *A. precatorius* leaves using GC-MS.

Retention time (min)	Name of compound	Area (%)
	**Phenolic compounds**	
6.246	Phenol	0.22
8.984	4-Vinylguaiacol	0.72
9.201	Syringol	0.95
9.831	Methylparaben	0.34
10.328	4-methyl-2,5-dimethoxybenzaldehyde	1.21
	**Steroids**	
16.805	β-Sitosterol	0.48
	**Terpenoids**	
8.872	Indolizine	0.25
	**Others**	
7.681	Butanedioic acid, hydroxy-,dimethyl ester	0.60
8.185	Methyl salicylate	0.10
8.396	Coumaran	2.35
9.467	1-(3,6,6-Trimethyl-1,6,7,7a-tetrahydrocyclopenta[c]pyran-1-y)ethanone	0.28
10.734	Cyclopentaneacetic acid, 3-oxo-2-pentyl-, methyl ester	0.91
11.210	2-Propenoic acid,3-(4-hydroxyphenyl)-, methyl ester	2.58
11.862	Hexadecanoic acid, methyl ester	2.08
13.948	2-Monopalmitin	2.82
15.054	Cyclotetracosane	3.00
15.601	γ-Tocopherol	0.80
15.706	Cyclooctacosane	1.12
15.902	Vitamin E	0.55
16.574	Triacontyl acetate	2.01
17.764	1-Octacosanol	0.07

**TABLE 4:** Compounds identified in the hexane extract (prepared via Soxhlet extraction) of *A. precatorius* leaves using GC-MS.

Retention time (min)	Name of compound	Area (%)
	**Phenolic compounds**	
11.870	Phenol, 3-isopropoxy-5-methyl	0.05
	**Terpenoids**	
10.722	Dihydroactinidiolide	0.12
12.003	Neophytadiene	1.54
11.814	(−)-Loliolide	0.35
18.151	Alnulin	0.23
	**Steroids**	
17.437	Campesterol	0.33
19.152	Stigmast-4-en-3-one	0.08
	**Others**	
2.621	Octane	0.10
4.924	Nonane	1.00
10.57	Dodecanoic acid	0.05
10.834	3-Mercapto-2(1H)-pyridinone	0.04
11.660	Myristic acid	0.08
11.682	1-Methylbicyclo(6.3.0)undec-5-en-9-one	0.09
12.185	3,7,11,15-Tetramethyl-2-hexadecen-1-ol	0.57
12.354	Hexadecanoic acid, methyl ester	0.05
12.564	Hexadecanoic acid	5.63
13.031	2-Pentadecanone,6,10,14-trimethyl-	0.11
13.047	9,12,15-Octadecatrienoic acid, methyl ester	0.18
13.096	Octadecanoic acid	5.24
14.477	2-Monopalmitin	0.23
15.035	9,12,15-Octadecatrienoic acid	0.18
15.077	Octadecanoic acid,2-hydroxy-1-( hydroxymethyl)ethyl ester	0.35
15.378	Cyclooctacosane	0.13
15.792	Bicyclo (10.8.0)eicosane, (E)-	0.37
16.415	Stigmasta-5,22-dien-3-ol, acetate, (3.beta.,22Z)-	0.46
16.541	1-Eicosanol	10.53
16.744	Vitamin E	0.37
18.067	(23S)-ethylcholest-5-en-3.beta.-ol	0.49
18.403	1-Tetracosanol	0.69
18.788	Oxirane, hexadecyl-	15.72

**TABLE 5:** Compounds identified in the ethyl acetate extract (prepared via Soxhlet extraction) of *A. precatorius* leaves using GC-MS.

Retention time (min)	Name of compound	Area (%)
	**Phenolic compounds**	
8.531	Benzoic acid	0.30
8.860	4-vinyl-phenol	3.26
9.834	2-Methoxy-4-vinylphenol	0.99
15.75	Naringenin	0.91
16.688	3-Methoxy-4,5,7-trihydroxyflavone	0.43
17.479	Cirsimaritin	4.48
	**Terpenoids**	
10.722	Dihydroactinidiolide	0.54
11.822	(−)-Loliolide	2.16
12.011	Neophytadiene	32.56
13.089	Phytol	1.71
	**Steroids**	
17.99	β-Sitosterol	0.64
	**Others**	
2.705	2-Propenoic acid, 2-methyl-,methyl ester	0.41
3.657	2-Pentanone,4-hydroxy-4-methyl-	0.35
8.089	2-Pentene,(Z)-	0.46
8.713	Benzoic acid, 2-hydroxy-, methyl ester	0.12
8.958	1H-Pyrrole-2,5-dione,3-ethyl-4-methyl-	0.25
9.378	Benzonitrile, 2-methyl-	0.19
9.735	Naphtalene,1,2-dihydro-1,1,6-trimethyl-	0.26
9.973	1-(3,6,6-Trimethyl-1,6,7,7a-tetrahydrocyclopentan(c)pyran-1-yl)ethanone	0.22
10.561	2,5-Cyclohexadiene-1,4-dione,2,6-bis(1,1-dimethyl)-	0.57
10.750	Dodecanoic acid	0.57
10.820	2,6-Dimethyl-3-(methoxymethyl)-p-benzoquinone	1.16
11.660	Myristic acid	2.30
11.569	1,2-Benzenediol,3,5-bis(1,1-dimethylethyl)-	0.40
11.709	1-Methylbicyclo(6.3.0)undec-5-en-9-one	1.05
12.508	Hexadecanoic acid	4.76
12.620	Ethyl palmitate	0.22
12.872	Margaric acid	0.19
13.201	Linolenic acid	5.59
13.264	Stearic acid	2.97
13.362	Ethyl stearate	0.25
14.363	Heptacosanol	0.41
15.028	Nonanoic Acid,9-(3-Hexenylidenecyclopropylidene)-	1.71
15.351	4,5′-Dihydroxy-7-methoxyflavanone	0.44
15.841	Stigmastan-6,22-ien,3,5-dehydro-	0.29
16.408	Stigmastan-3,5,22-trien	0.90
16.744	Vitamin E	0.76

**TABLE 6:** Compounds identified in the methanol extract (prepared via Soxhlet extraction) of *A. precatorius* leaves using GC-MS.

Retention time (min)	Name of compound	Area (%)
	**Phenolic compounds**	
6.787	Phenol	0.09
8.867	4-vinylphenol	12.18
9.483	2-Methoxy-4-vinylphenol	0.71
10.820	4-vinyl-syringol	0.37
17.472	Cirsimaritin	0.53
	**Terpenoids**	
11.822	(−)-Loliolide	1.59
11.941	Neophytadiene	12.18
13.089	Phytol	0.31
	**Steroids**	
17.598	Stigmasterol	0.54
	**Others**	
2.545	2-Furancarboxaldehyde	0.22
5.555	Butyrolactone	0.08
5.856	2-Hydroxy-2-cyclopenten-1-one	0.80
7.928	Benzoic acid, methyl ester	0.56
8.159	Octanoic acid, methyl ester	0.13
8.783	Benzoic acid,2-methyl-, methyl ester	0.17
9.735	Capric acid	0.29
10.568	Lauric acid, methyl ester	0.22
10.764	Dodecanoic acid	1.16
11.506	Tetradecanoic acid, methyl ester	3.02
11.660	Myristic acid	2.77
12.032	2-Pentadecanone	1.32
13.040	Methyl,8,11,14-heptadecatrienoate	0.51
13.187	Linolenic acid	0.55
13.25	Stearic acid	0.56
13.53	Methanone, (4-chlorophenyl)(4-hydroxyphenyl)-	0.11
14.440	Hexadecanoic acid, 2-hydroxy-1-(hydroxymethyl)ethyl ester	0.44
15.07	Octadecanoic acid,2-hydroxy-1-(hydroxymethyl)ethyl ester	0.37
15.652	4-amino-5-tert-butyl-4′-(dimethylamino)biphenyl-3-carbonitrile	0.24
15.813	Glycerol tricaprylate	0.43

**TABLE 7:** IC_50_ values of maceration-based extracts of *A. precatorius* leaves against selected normal and cancer cell lines (μg/mL).

Cell lines	Hexane extract (Maceration)	Ethyl acetate extract (Maceration)	Methanol extract (Maceration)
HeLa (cervix)	325.0 ± 38.4	371.0 ± 52.0	325 ± 12.2
MCF7 (breast)	425.5 ± 75.7	>495.0	330 ± 37.0
MDA-MB-231 (breast)	80.75 ± 64.0	206.50 ± 9.2	254.5 ± 57
SW 480 (colon)	301.3 ± 39.1	447.5 ± 31.8	350.3 ± 28
MCF-10a (normal breast)	> 495	> 495	> 495
NIH (3T3) (normal fibroblast)	> 495	> 495	> 495

*Note*: Data are expressed as mean ± SD from three independent experiments (triplicates).

## APPENDIX

### Proliferation Growth Curve

#### (a) HeLa cells

SUPPLEMENTARY FIGURE 1 Anti-proliferative activity of *A. precatorius* successive (maceration) hexane-, ethyl acetate- and methanol- leaves extracts on HeLa cells. *Note*: The IC_50_ obtained for hexane extract was 325 μg/mL, ethyl acetate extract was 371 μg/mL and methanol extract was 352 μg/mL. The results were expressed as mean, ± SD of three independent experiments with three replicates.

#### (b) MCF-7 cells

SUPPLEMENTARY FIGURE 2 Anti-proliferative activity of *A. precatorius* successive (maceration) hexane-, ethyl acetate- and methanol- leaves extracts on MCF-7 cells. *Note*: The IC_50_ obtained for hexane extract was 672 μg/mL and methanol extract was 423 μg/mL. While ethyl acetate extract was > 495 μg/mL. The results were expressed as mean, ± SD of three independent experiments with three replicates.

#### (c) MDA-MB-231 cells

SUPPLEMENTARY FIGURE 3 Anti-proliferative activity of *A. precatorius* successive (maceration) hexane-, ethyl acetate- and methanol- leaves extracts on MDA-MB-231 cells. *Note*: The IC_50_ obtained for hexane extract was 80.75 μg/mL, ethyl acetate extract was 207 μg/mL and methanol was 255 μg/mL. The results were expressed as mean, ± SD of three independent experiments with three replicates.

#### (d) SW480 cells

SUPPLEMENTARY FIGURE 4 Anti-proliferative activity of *A. precatorius* successive (maceration) hexane-, ethyl acetate- and methanol- leaves extracts on SW480 cells. *Note*: The IC_50_ obtained for hexane extract was 301.3 μg/mL, ethyl acetate extract was 447.5 μg/mL and methanol was 350.3 μg/mL. The results were expressed as mean, ± SD of three independent experiments with three replicates.

#### (e) MCF10a cells

SUPPLEMENTARY FIGURE 5 Anti-proliferative activity of *A. precatorius* successive (maceration) hexane-, ethyl acetate- and methanol- leaves extracts on MCF10a cells. *Note*: No IC_50_ was obtained even at the maximum concentration of 495 μg/mL. The results were expressed as mean, ± SD of three independent experiments with three replicates.

#### (f) NIH(3T3) cells

SUPPLEMENTARY FIGURE 6 Anti-proliferative activity of *A. precatorius* successive (maceration) hexane-, ethyl acetate- and methanol- leaves extracts on NIH(3T3) cells. No IC_50_ was obtained even at the maximum concentration of 495μg/ml. The results were expressed as mean, ± SD of three independent experiments with three replicates.
