# Supplementary figures and images for: Phytoconstituents Analysis and In Vitro Antiproliferative Activity of Abrus precatorius Leaves on Cancer Cells
Source: Trop Life Sci Res. 2026 Mar 31;37(1):241–71. doi: 10.21315/tlsr2026.37.1.12 (PMC13128000; doi:10.21315/tlsr2026.37.1.12)

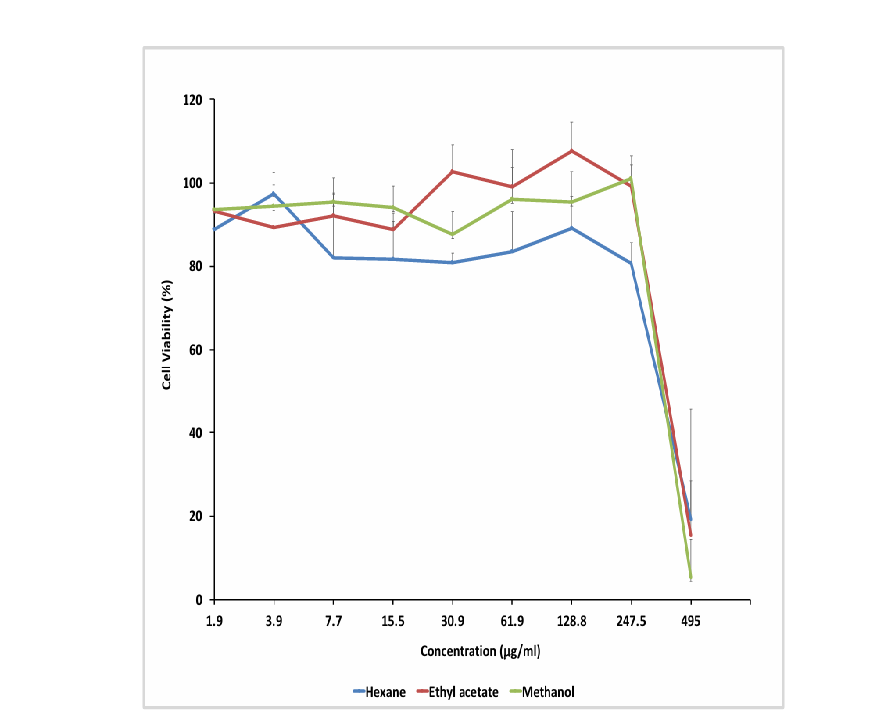

Supplement: SUPPLEMENTARY FIGURE 1 — Anti-proliferative activity of A. precatorius successive (maceration) hexane-, ethyl acetate- and methanol- leaves extracts on HeLa cells. Note: The IC50 obtained for hexane extract was 325 μg/mL, ethyl acetate extract was 371 μg/mL and methanol extract was 352 μg/mL. The results were expressed as mean, ± SD of three independent experiments with three replicates. [file TLSR_37-1-241-g00S1.tif]

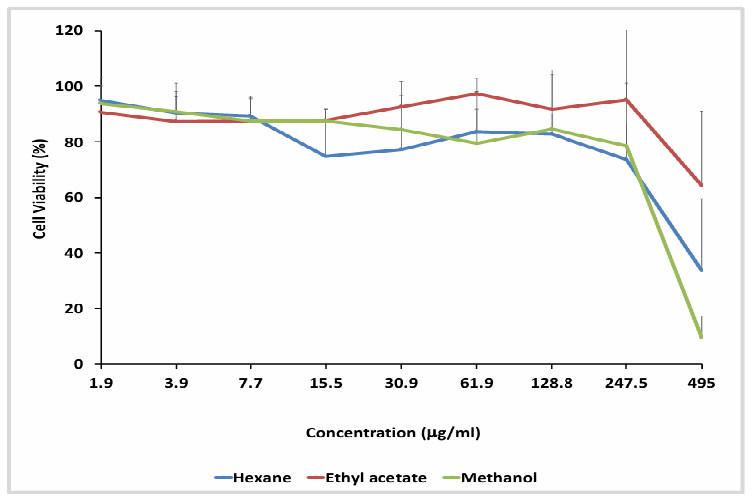

Supplement: SUPPLEMENTARY FIGURE 2 — Anti-proliferative activity of A. precatorius successive (maceration) hexane-, ethyl acetate- and methanol- leaves extracts on MCF-7 cells. Note: The IC50 obtained for hexane extract was 672 μg/mL and methanol extract was 423 μg/mL. While ethyl acetate extract was > 495 μg/mL. The results were expressed as mean, ± SD of three independent experiments with three replicates. [file TLSR_37-1-241-g00S2.tif]

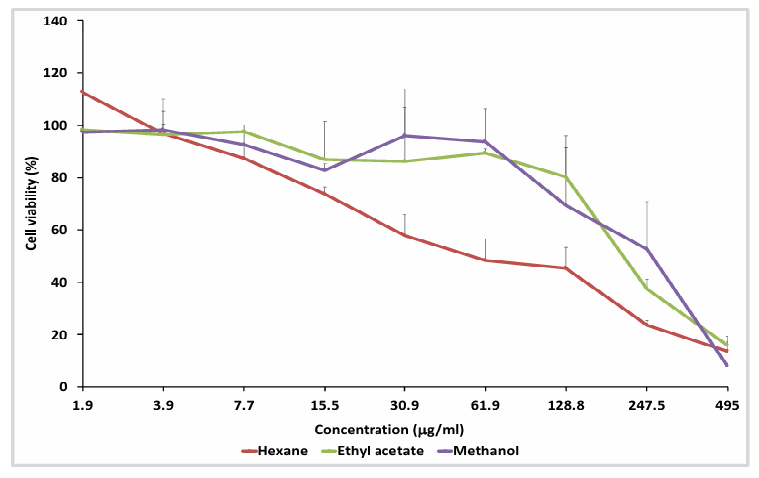

Supplement: SUPPLEMENTARY FIGURE 3 — Anti-proliferative activity of A. precatorius successive (maceration) hexane-, ethyl acetate- and methanol- leaves extracts on MDA-MB-231 cells. Note: The IC50 obtained for hexane extract was 80.75 μg/mL, ethyl acetate extract was 207 μg/mL and methanol was 255 μg/mL. The results were expressed as mean, ± SD of three independent experiments with three replicates. [file TLSR_37-1-241-g00S3.tif]

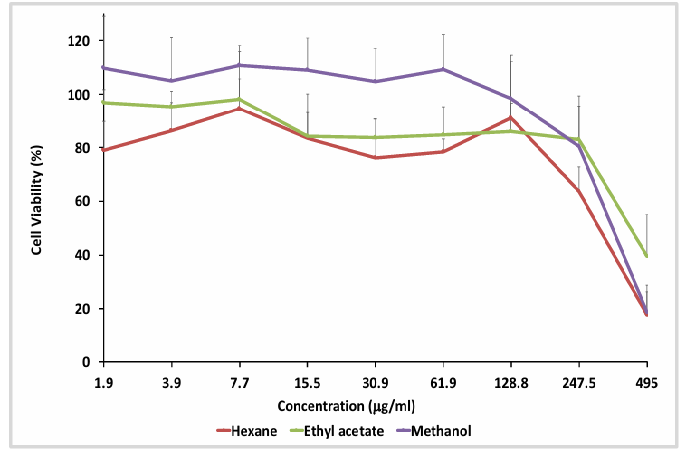

Supplement: SUPPLEMENTARY FIGURE 4 — Anti-proliferative activity of A. precatorius successive (maceration) hexane-, ethyl acetate- and methanol- leaves extracts on SW480 cells. Note: The IC50 obtained for hexane extract was 301.3 μg/mL, ethyl acetate extract was 447.5 μg/mL and methanol was 350.3 μg/mL. The results were expressed as mean, ± SD of three independent experiments with three replicates. [file TLSR_37-1-241-g00S4.tif]

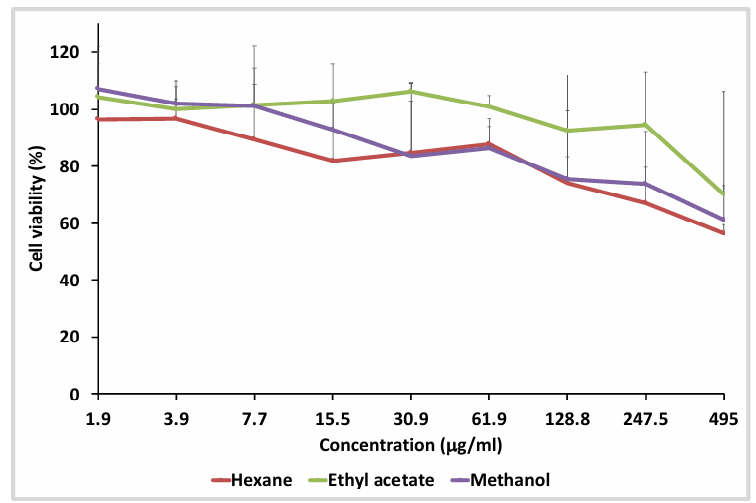

Supplement: SUPPLEMENTARY FIGURE 5 — Anti-proliferative activity of A. precatorius successive (maceration) hexane-, ethyl acetate- and methanol- leaves extracts on MCF10a cells. Note: No IC50 was obtained even at the maximum concentration of 495 μg/mL. The results were expressed as mean, ± SD of three independent experiments with three replicates. [file TLSR_37-1-241-g00S5.tif]

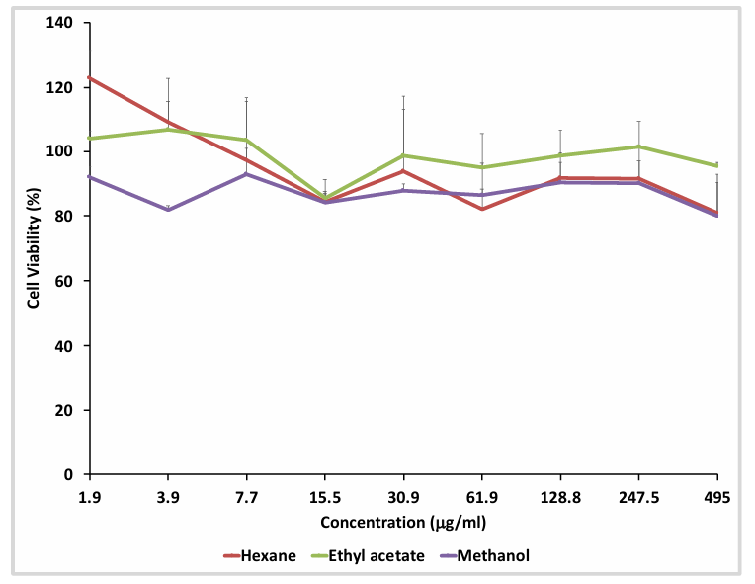

Supplement: SUPPLEMENTARY FIGURE 6 — Anti-proliferative activity of A. precatorius successive (maceration) hexane-, ethyl acetate- and methanol- leaves extracts on NIH(3T3) cells. No IC50 was obtained even at the maximum concentration of 495μg/ml. The results were expressed as mean, ± SD of three independent experiments with three replicates. [file TLSR_37-1-241-g00S6.tif]
